# Molecular hydrogen: a potential guardian against environmental radiation damage

**DOI:** 10.4103/mgr.MEDGASRES-D-25-00163

**Published:** 2026-09-12

**Authors:** Yusuke Ichikawa, Bunpei Sato, Yoshiyasu Takefuji, Fumitake Satoh

**Affiliations:** 1 Research and Development Department, MiZ Company Limited, Kanagawa, Japan; 2 Faculty of Data Science, Musashino University, Tokyo, Japan; 3 Keio University, Tokyo, Japan

**Keywords:** aging, environmental radiation, hydrogen, hydroxyl radical, oxidative damage, oxidative stress, radiation resistance, senescent cells

## Abstract

Molecular hydrogen is known to selectively convert hydroxyl radicals into harmless water and thereby mitigating oxidative stress. Due to hydrogen molecules have masses comparable to protons and neutrons, can also decelerate neutrons and protons present in environmental radiation, thus exerting a physical shielding effect. Through this dual action, chemical radical scavenging and physical radiation shielding, hydrogen may protect cellular integrity and potentially promote biological longevity. In contrast, hydrogen can intervene at the stage of preventing biomolecular damage induced by environmental radiation and provide comprehensive protection at the tissue and cellular levels, uniquely minimizing radiation-induced damage. This review systematically summarizes existing knowledge regarding the antioxidant and radioprotective effects of hydrogen, discusses its role in mitigating oxidative stress and protecting cells from environmental radiation, and proposes a novel hypothesis that hydrogen has universally protected from radiation-induced damage, highlighting its potential for promoting biological longevity.

## Introduction

Humans are continuously exposed not only to artificial radiation such as X-rays used in medical settings but also to environmental radiation from cosmic rays and radioactive materials present in soil and building materials.^1^,^2^ Although the environmental radiation dose on Earth is relatively low, making its effects imperceptible in daily life, cosmic rays pose serious health risks for airline pilots and astronauts.^3^ Indeed, pilots’ flight hours are regulated, and astronauts exhibit accelerated aging phenomena during space missions, suggesting that even low-dose environmental radiation on Earth may gradually induce cellular damage and accelerate aging over time.

Cellular damage induced by environmental radiation can occur via two mechanisms. The first is direct damage caused by the energy of the radiation itself. The second arises indirectly when radiation interacts with water molecules within the body, generating highly reactive hydroxyl radicals (·OH). These radicals are potent oxidizing agents capable of inducing irreversible damage to DNAs, lipids, and proteins.^4^ Since complete avoidance of daily exposure is unrealistic, effective defense strategies require both shielding from radiation and mechanisms to efficiently eliminate hydroxyl radicals generated within the body. Ideally, a radioprotective agent should possess both the ability to attenuate radiation itself and to scavenge hydroxyl radicals formed secondarily by radiation exposure.

Recently, molecular hydrogen has attracted attention in the medical field due to its unique ability to convert hydroxyl radicals into harmless water.^5^ Hydrogen’s extremely small molecular size allows it to easily penetrate cell membranes and organelles, reaching widespread regions within the body and efficiently mitigating locally generated oxidative stress.^5^ Moreover, hydrogen may reduce the kinetic energy of heavy ions and neutrons, suggesting potential radioprotective effects both *in vivo* and *ex vivo*.^6^ Thus, hydrogen may provide dual protection against radiation-induced oxidative stress and direct radiation attenuation, representing a promising strategy for aging prevention and health maintenance.

Currently, reductionist approaches focus only on particular aspects of aging and may be insufficient for maintaining overall cellular and tissue homeostasis. In contrast, hydrogen can directly eliminate hydroxyl radicals generated by radiation, preserving cells in a minimally damaged state and offering comprehensive protection to support the integrity of the entire organism. Even in the context of pursuing immortality, hydrogen presents a distinct and potentially complementary strategy to existing interventions.

In this review, we systematically summarize current knowledge regarding the antioxidant and radioprotective effects of hydrogen and discuss its role in mitigating radiation-induced oxidative stress. Furthermore, we explore potential mechanisms through which hydrogen may contribute to biological longevity and provide insight into the fundamental challenge that all living organisms inevitably face, aging and death, thereby deepening our understanding of the feasibility of extending health and life spans.

## Methods

During the preparation of this manuscript, we conducted a literature search using PubMed, Web of Science, and Google Scholar for articles published up to July 2025. The search employed combinations of keywords including “hydrogen,” “molecular hydrogen,” “oxidative stress,” “radiation,” “cosmic radiation,” “hydroxyl radical,” “radioresistant bacteria,” and “anti-aging.” We included peer-reviewed articles in English that focused on the effects of hydrogen on oxidative stress, radiation-induced damage, and aging, while excluding non-peer-reviewed studies (e.g., abstracts), irrelevant research, and articles lacking mechanistic data. Additionally, reference lists of relevant articles were screened to identify further pertinent studies.

## Hydroxyl Radical Is a Source of Cellular Damage

The degradation of cellular components progresses primarily due to hydroxyl radicals. Just as automobiles generate power by combusting gasoline with oxygen in their engines, biological cells produce energy through mitochondrial respiration, in which oxygen is used to oxidize sugar metabolites derived from food.^7^ This mitochondrial process is referred to as “respiration.” Over time, an automobile engine accumulates soot from the incomplete combustion of fuel, leading to mechanical deterioration. Similarly, incomplete oxidation in mitochondria, the “engines” of cells, can result in the formation of hydroxyl radicals, which in turn contribute to mitochondrial damage.

Within mitochondria, hydroxyl radicals are generated via the Fenton reaction, in which hydrogen peroxide-produced during electron and proton transfer to oxygen, is reduced by transition metal ions such as ferrous iron (Fe^2+^) (Equation 1).^8^ Hydroxyl radicals can also be produced by the radiolysis of water molecules, triggered by ionizing radiation (Equation 2).^9^ As mammalian cells contain a high proportion of intracellular water, exposure to radiation results in the generation of hydroxyl radicals within cells. The decline in mitochondrial function and the induction of genomic instability following exposure to radiation, such as X-rays, are primarily attributed to hydroxyl radicals formed through the radiolysis of intracellular water.^10^

H_2_O_2_ + Fe^2+^ → ·OH + OH^–^ + Fe^3+^      (1)

H_2_O → ·H_2_O^+^ + e^–^      (2)

·H_2_O^+^ → H^+^ + ·OH

·H_2_O^+^ + H_2_O → H_3_O^+^ + ·OH

## Environmental Radiation

In fact, radiation in our daily lives is not limited to that emitted from medical devices used in radiation therapy. One significant source is cosmic rays originating from outer space.^1^ For example, astronauts aboard the International Space Station are exposed to low-dose cosmic radiation of 0.5 to 1.0 mSv per day.^2^ By contrast, people on Earth are exposed to an average of about 2.4 mSv per year from natural background radiation, according to authoritative evaluations by United Nations Scientific Committee on the Effects of Atomic Radiation^11^ and International Commission on Radiological Protection.^12^ Although primary cosmic rays are largely blocked by the Earth’s atmosphere and rarely reach the surface, secondary radiation generated through interactions between cosmic rays and atmospheric particles continuously penetrates our bodies. Therefore, while the atmosphere does provide a shielding effect against cosmic radiation, this protection is not complete.^13^

In addition to cosmic sources, we are also exposed to terrestrial radiation from naturally occurring radioactive materials found in soil.^14^ Even building materials contain trace amounts of these radioactive elements, and we are constantly exposed to radiation emitted from walls and roofs while indoors. In this paper, we refer to such naturally occurring background radiation as environmental radiation.

Environmental radiation can be visualized using a simple experimental apparatus known as a cloud chamber, invented by the Scottish physicist Charles Thomson Rees Wilson.^15^ Demonstrations of the cloud chamber are widely available in video format online, and they provide a vivid impression of the extent of everyday radiation exposure. It is well established that the aging process, which proceeds gradually on Earth, is accelerated in astronauts in space due to increased exposure to cosmic radiation.^3^ Although the quantity of hydroxyl radicals generated by environmental radiation is generally lower than that produced during normal respiration, increases in reactive oxygen species (ROS), such as superoxide and hydrogen peroxide, have been observed in mitochondria following radiation therapy.^16^ In other words, because the human body is composed of approximately 60% water, exposure to environmental radiation can generate hydroxyl radicals through the radiolysis of intracellular water molecules.^3^ In dry environments where water molecules are scarce, hydroxyl radicals are less likely to form. As a result, organisms such as *Milnesium tardigradum* (tardigrades) and *Polypedilum vanderplanki* (sleeping chironomids), which can survive in anhydrobiotic (dry) states, exhibit remarkable resistance to high levels of radiation.^17^,^18^ Their tolerance is attributed to the reduced availability of intracellular water molecules, which suppresses the generation of hydroxyl radicals by radiolysis. In contrast, in organisms like humans, where cells contain abundant water, hydroxyl radicals produced by environmental radiation can form in close proximity to chromosomal DNA. These radicals can attack the carbon atoms of deoxyribose, leading to strand breaks in DNA. When such breaks occur in tumor suppressor genes, the resulting mutations may disrupt cell cycle control and contribute to carcinogenesis.^19^ If the cumulative cellular damage caused by hydroxyl radicals targeting DNA and other biomolecules underlies both aging and intractable diseases, then eliminating hydroxyl radicals generated within cells could be a promising strategy for preventing aging and associated diseases (**Figure 1**). The subsequent section further develops these ideas by examining the long-term disruption of biological homeostasis.

**Figure 1:**
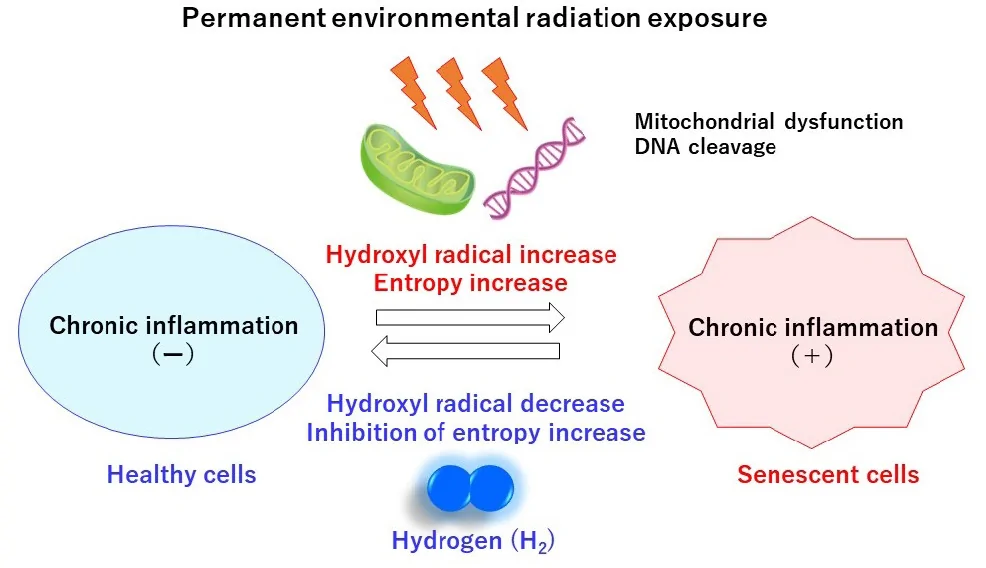
Accumulation of mitochondrial dysfunction and DNA damage caused by environmental radiation accelerates aging. Hydrogen inhibits mitochondrial dysfunction and DNA damage by scavenging hydroxyl radicals, preventing aging and restoring cells to a normal state. Created with Microsoft PowerPoint 2019.

## Long-Term Exposure to Environmental Radiation Disrupts Biological Homeostasis

SpaceX, led by Elon Musk, has proposed a long-term plan for human colonization of Mars. However, one of the major obstacles to this endeavor is the presence of intense cosmic radiation in space, which poses serious health risks to astronauts.^20^ Even without leaving Earth, exposure to secondary cosmic radiation that penetrates the atmosphere has been associated with increased incidences of cancer, myocardial infarction, congenital abnormalities, and higher mortality rates.^3^ Over the course of Earth’s biological history, secondary cosmic radiation has contributed to mass extinction events and has likely increased the frequency of DNA mutations on a geological time scale.

A study reported a statistically significant correlation between total mortality and secondary cosmic radiation exposure among the population of São Paulo, Brazil.^21^ Although the human body possesses homeostatic feedback mechanisms to respond to external stressors, it remains highly vulnerable to the uncontrolled hydroxyl radicals generated by the radiolysis of intracellular water triggered by secondary radiation. These radicals are chemically aggressive and indiscriminately attack biomolecules, including DNA, without any regulatory control.

Despite the existence of a controversial hypothesis known as “radiation hormesis,” which suggests that low doses of radiation might have beneficial stimulatory effects akin to a mild toxin.^22^ However, we think that this is the result of cell and animal studies, and the benefit to humans has not been confirmed to our knowledge. The rationale is that even low-dose radiation exposure can produce hydroxyl radicals that cause stochastic and potentially harmful damage to cellular components, leading to unpredictable biological consequences. For example, adverse side effects of radiation therapy for cancer arise because the irradiation damages not only tumor cells but also the surrounding healthy tissue. This is because even normal cells can be killed by small amounts of radiation leaking around cancer cells. Even with advancements in precision radiotherapy, it remains extremely difficult to fully eliminate collateral radiation damage—except through one promising method currently known: the selective scavenging of hydroxyl radicals by molecular hydrogen, which has been shown to neutralize hydroxyl radical without interfering with other essential ROS.

The concept of “pre-symptomatic disease” refers to a health condition in which pathological processes are ongoing in the body despite the absence of observable symptoms.^23^ We hypothesize that long-term exposure to environmental radiation may gradually erode physiological homeostasis, thereby initiating a pre-symptomatic state that could eventually progress into full-blown, intractable diseases.

## Ideas Necessary for the Challenge of Immortality

In recent years, there has been fierce competition in the development of senolytic drugs that attempt to eliminate senescent cells with pharmaceuticals.^24^ The basic drug discovery concept of these senolytic drugs is to eliminate senescent cells by targeting and inhibiting or activating specific gene products inside or outside the cell. It is important to note that drugs are always associated with side effects, and the attempt to eliminate senescent cells with senolytic drugs implies that the senolytic drugs are toxic to the cells in some way. For example, piperlongumine, an alkaloid extracted from Piper longum, attempts to eliminate senescent cells by increasing cellular ROS.^25^ Piperlongumine decreases the expression level of oxidation resistance 1, an important antioxidant protein that regulates the expression of various antioxidant enzymes.^26^ However, since suppression of oxidation resistance 1 expression increases intracellular ROS levels, it is questionable whether lowering the level of response proteins with piperlongumine can really suppress the progression of aging.

The key idea in controlling the aging process is not to “eliminate aging cells” but to “maintain young cells.” If hydroxyl radicals are the substances that accelerate the aging process, then it may be necessary to construct an environment in which hydroxyl radicals can be more safely removed and young cells that are less damaged can remain young cells.

## The Smallest Diatomic Molecule—Hydrogen

Our bodies are exposed to a constant barrage of environmental radiation, which is causing a gradual aging and unwellness process. Once environmental radiation penetrates the body and reacts with water molecules in the body, uncontrollable hydroxyl radicals are generated, and there is nothing we can do about it. Hydrogen is the guardian deity that scavenges hydroxyl radicals in the body.^27^ Many papers have already reported that hydrogen can ameliorate radiation damage in humans and animals.^28^,^29^ We presents two of its main mechanisms from a different perspective.

The first is that hydrogen is the only substance capable of reacting with hydroxyl radicals, which are produced by radiation penetrating the living organism and breaking down the water molecules in the cells, and converting them to water molecules (**Figure 2**).^5^ The hydroxyl radical is an extremely reactive species characterized by the presence of a single unpaired electron on an oxygen atom. It is considered one of the most potent oxidizing ROS in biological systems.^4^ Due to its high reactivity, hydroxyl radical can rapidly initiate hydrogen abstraction reactions even from stable molecules with covalent bonds, thereby inducing immediate and non-selective damage to vital biomolecules, such as membrane lipids, DNAs, and proteins.

**Figure 2:**
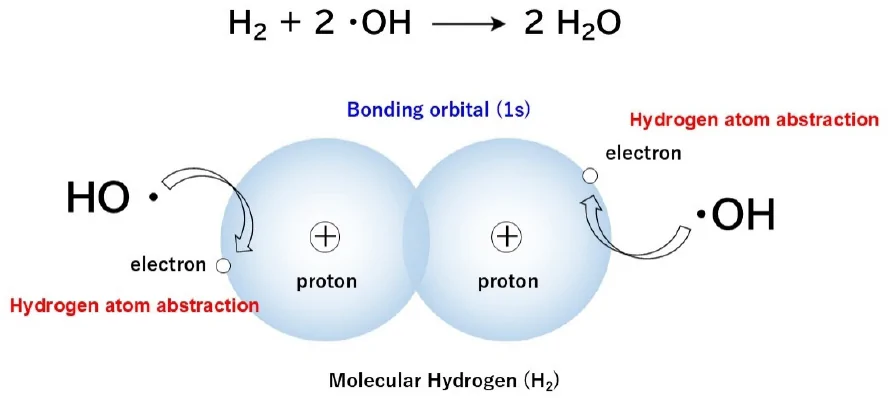
Reaction of H_2_ with hydroxyl radicals. Hydrogen molecules form a stable covalent bond by sharing two electrons in the 1s orbital. However, because the bond dissociation energy of the H–H bond is not particularly high, hydrogen is susceptible to abstraction reactions by radicals. Created with Microsoft PowerPoint 2019. H_2_: Molecular hydrogen; H_2_O: water.

In contrast, molecular hydrogen is a relatively stable diatomic molecule formed by the sharing of two electrons between two hydrogen atoms through a bonding molecular orbital (σ1s).^30^ The H–H bond has a bond dissociation energy of approximately 436 kJ/mol, making it stable under normal conditions. However, it can react with highly reactive radical species such as the hydroxyl radical. Specifically, hydroxyl radical can approach one of the hydrogen atoms in a hydrogen molecule, abstract it, and in the process, be converted into water (**Figure 2**).

This reaction is regarded as one of the potential pathways for the detoxification of hydroxyl radical in vivo. It is desirable that hydroxyl radicals be selectively scavenged by molecular hydrogen before they irreversibly react with critical cellular components such as DNA or lipids. Therefore, the adequate delivery of molecular hydrogen within the body may represent an important strategy for mitigating oxidative stress.^5^,^31^

In addition, hydrogen, the smallest diatomic molecule, can easily penetrate cell membranes and reach cells and intracellular organelles throughout the body, from inside the brain to the tips of the feet. Therefore, it can protect against hydroxyl radicals generated not only by respiration inside cells throughout the body, but also by environmental radiation that falls on the whole body, thus contributing to keeping young cells young. If the aging process is the causative agent that increases cell entropy due to hydroxyl radicals, then hydrogen can scavenge hydroxyl radicals to keep cells in as steady a state as possible and inhibit the increase in cell entropy due to hydroxyl radicals (**Figure 2**).

Hydrogen converts hydroxyl radicals produced by radiation water splitting into water molecules, and can show excellent ameliorating effects on radiation damage in irradiated cells and animal models.^28^ For example, hydrogen can improve the quality of life of liver cancer patients treated with radiotherapy and reduce hematopoietic disorders in end-stage cancer patients treated with intensity modulated radiation therapy.^29^,^32^

The second mechanism is that hydrogen itself has a shielding effect that slows down the heavy ions of galactic cosmic radiation, protons of solar energetic particles, and neutrons formed when galactic cosmic radiations and solar energetic particles react with materials, and thus may also shield the radiation that penetrates the cells inside the body, preventing radiation from destroying the materials that make up the body.^6^ The high efficacy of hydrogen in radiation shielding arises from its mass being close to that of neutrons and protons, which maximizes energy transfer during collisions and effectively decelerates incident radiation. In contrast, heavier nuclei exhibit lower energy transfer, resulting in limited shielding effectiveness. Consequently, materials with high hydrogen content are the most effective for radiation shielding. Materials for shielding against environmental radiation are being developed in the space program, and the materials being employed for this are those that contain a large number of hydrogen atoms.^33^ In particular, molecular hydrogen does not readily decompose upon irradiation and does not produce secondary radiation, making it an ideal radioprotective agent.^33^ In the next section, we propose a hypothesis that supports the hypothesis that hydrogen generated in the body would shield against environmental radiation and contribute to biological defense against radiation.

## A New Hypothesis on the Mechanisms of Radiation Resistance in Radioresistant Bacteria

Radioresistant bacteria are bacteria that exhibit resistance to radiation. The first radioresistant bacterium was discovered in canned food sterilized by γ-rays and named *Deinococcus radiodurans*. Since then, many other types of radioresistant bacteria have been identified.^34^,^35^ Radioresistant bacteria also exhibit resistance to hydrogen peroxide.^36^ Several hypotheses have been proposed to explain this resistance, including the involvement of a group of genes called Rec related to DNA repair,^37^ manganese complexes,^38^ and heat shock proteins.^39^ However, none of these theories seem to fully explain the mechanisms of radio-resistance. The radio-resistance potential of *Deinococcus radiodurans* has been thought to depend on DNA protection from radiation-induced DNA damage due to DNA repair, but it is now clear that protection from radiation-induced oxidative damage of proteins is more important than DNA protection.^38^

Along with *Deinococcus radiodurans*, hyperthermophilic archaea, which are strictly anaerobic and live deep in the sea and in hot springs, are also known to be resistant not only to heat but also to radiation.^39^,^40^,^41^,^42^,^43^ Hydrogen plays a central role in microbial systems, much like the central position hydrogen occupies in the periodic table of elements when focusing on biological functions.^44^ At a time before oxygen existed in Earth’s atmosphere, primitive microorganisms produced energy by utilizing hydrogen dissolved in seawater, which originated from reactions between hydrothermal fluids and minerals in the mantle. Primitive microorganisms possess hydrogenase enzymes to metabolize hydrogen, and *Deinococcus radiodurans* also contains hydrogenase.^45^ Two types of hydrogenases are known: those with a dinuclear iron [FeFe] active site, and those with a dinuclear iron-nickel [FeNi] active site. Hydrogenases catalyze reversible redox reactions involving hydrogen; these enzymes can both decompose hydrogen and split water to produce hydrogen (Equation 3).^46^ Primitive life generated energy by utilizing hydrogen in hydrogen-rich environments and by reducing protons to produce molecular hydrogen in hydrogen-depleted environments. It can be said that primitive life existed in an environment constantly surrounded by hydrogen, both intracellularly and extracellularly.^47^


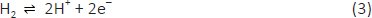


Because bacteria with hydrogenases are anaerobes that do not metabolize oxygen, they are not exposed to attacks by hydroxyl radicals, which are oxygen metabolites. We hypothesize that the radioresistance of these anaerobic bacteria stems from their resistance to hydroxyl radicals produced by radiation-induced water radiolysis. We propose the following hypothesis for the resistance of hyperthermophilic archaea and *Deinococcus radiodurans* from the seafloor against hydroxyl radicals: the hydrogen-rich environment shields these organisms from external radiation, and the hydrogen produced intracellularly by their hydrogenases rapidly converts hydroxyl radicals, generated by radiation-induced water radiolysis, back into water molecules, thereby protecting DNA and proteins from radiation damage.

Hydrogen-producing bacteria possessing hydrogenase genes, such as Bacteroides and Firmicutes, inhabit the human gut and are known to contribute to maintaining human health and inhibiting aging.^48^ Interestingly, *Deinococcus* species have also been found in animal intestines.^49^ Regarding *Deinococcus radiodurans*, surface glycans have been analyzed in the context of human health. However, the precise relationship between these glycans and human health remains insufficiently explored. If *Deinococcus radiodurans* contributes to human health, this may be elucidated by focusing on the hydrogenases common to these bacteria that metabolize hydrogen.

## Potential of Hydrogen Therapy for Mitigating Cosmic Ray-Induced Traumatic Brain Injury

In addition to cancer and cardiovascular diseases, the effects of cosmic radiation on the central nervous system are recognized as significant risks for astronauts. It is possible that environmental radiation from space penetrates the brain, generating hydroxyl radicals, which in turn accelerate the aging of neuronal cells and contribute to the development of neurodegenerative diseases such as Parkinson’s disease (**Figure 3**).^50^,^51^

**Figure 3:**
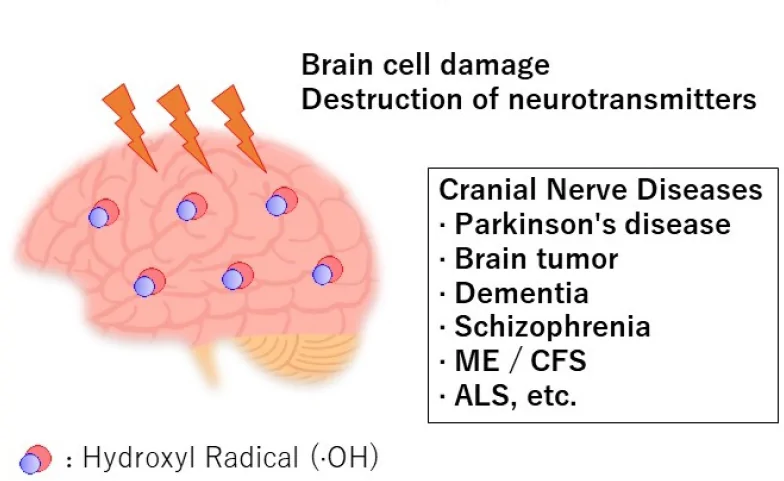
Environmental radiation destroys the central nervous system. Cosmic radiation poses a significant health risk to astronauts by generating hydroxyl radicals in the brain, accelerating neuronal aging, and increasing the risk of neurodegenerative diseases, such as Parkinson’s disease. Created with Microsoft PowerPoint 2019. ALS: Amyotrophic lateral sclerosis; ME/CFS: myalgic encephalomyelitis/chronic fatigue syndrome.

In cases of war or terrorism, individuals who survive blasts may still suffer brain damage due to shock waves, resulting in traumatic brain injury (TBI), which can lead to higher-order brain dysfunction.^52^ TBI is known to be caused not only by blast waves, but also by prolonged exposure of the brain to cosmic radiation, which may damage microglia, which are responsible for maintaining homeostasis in the central nervous system.^53^,^54^

Hydrogen is highly effective in suppressing cellular senescence in neurodegenerative diseases and may also contribute to the improvement of TBI.^55^ Hydrogen diffuses across the blood-brain barrier, reaches deep brain regions, and neutralizes hydroxyl radicals by converting them into water molecules. This process can mitigate severe DNA damage and oxidative stress in neural tissues, promote recovery from TBI, and reduce chronic inflammation. Regular inhalation of hydrogen gas may offer continuous protection for the brain against TBI caused by persistent environmental radiation exposure (**Figure 4**).

**Figure 4:**
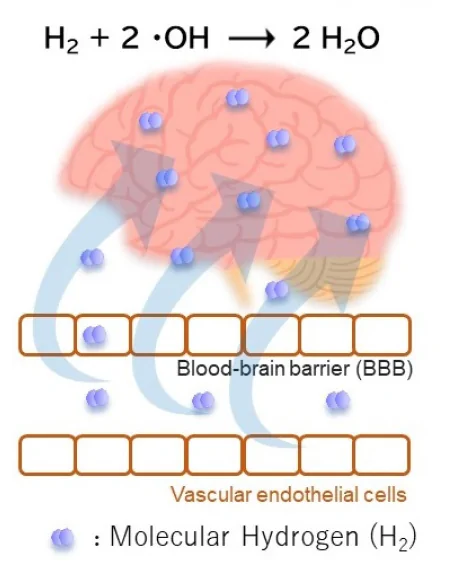
Hydrogen scavenges radiation-induced hydroxyl radicals in the brain. Hydrogen ingested by inhalation passes through the blood–brain barrier and reaches the brain, where it converts hydroxyl radicals (·OH) induced by environmental radiation into water molecules (H_2_O) in the brain, protecting the brain from environmental radiation. Created with Microsoft PowerPoint 2019.

Intestinal bacteria in the human gut may produce hydrogen that helps protect cells from environmental radiation; however, the quantity produced endogenously is insufficient to counteract radiation-induced aging. Therefore, exogenous hydrogen intake via inhalation is necessary to exert anti-aging effects.^5^

Hydrogen is drawing attention as a clean, next-generation energy source. Its cleanliness applies not only to energy applications but also to medical use.^56^ Just as hydrogen can replace gasoline or other carbon-based fuels in automobiles to prevent soot formation and extend engine life, it can also protect cellular “engines,” the mitochondria, by converting hydroxyl radicals into water molecules. This helps prevent mitochondrial deterioration, maintains the function of youthful cells, and ultimately contributes to cellular longevity.

## Conclusion

This review demonstrates that environmental radiation generates hydroxyl radicals through the radiolysis of intracellular water molecules, thereby promoting cellular damage and accelerating aging. Molecular hydrogen has the unique ability to selectively convert hydroxyl radicals into harmless water molecules and additionally attenuate or shield radiation itself, suggesting its dual role in radioprotection and anti-aging. Unlike conventional strategies focused on eliminating senescent cells, hydrogen offers a novel paradigm that emphasizes the preservation of young and functional cells in their pristine state.

Furthermore, evidence from radiation-resistant bacteria and hydrogen-metabolizing gut microbiota implies that hydrogen may have played a universal role in protecting life against radiation throughout evolution. Given its ability to freely diffuse into all organs and subcellular compartments, hydrogen also shows promise in mitigating neurological disorders, including traumatic brain injury and neurodegeneration. These findings extend beyond health span and disease prevention, providing potential applications to the challenges of human space exploration.

Introducing hydrogen at safe concentrations into the habitat atmosphere, thereby allowing continuous low-level inhalation, could serve as a rational strategy to mitigate oxidative stress and suppress radiation-induced cellular damage. This approach complements conventional external shielding methods and may provide a sustainable means of supporting human survival under extreme conditions.

In summary, molecular hydrogen should be regarded not merely as an antioxidant but as a “guardian molecule” that protects life from radiation.

**Facts**

1. Molecular hydrogen selectively removes hydroxyl radicals, thereby reducing oxidative stress.
2. Molecular hydrogen protects cellular integrity and may extend lifespan through a combination of chemical and physical mechanisms.
3. Molecular hydrogen can intervene at the stage of biomolecular damage caused by radiation, providing comprehensive protection to tissue and cells.

**Open Questions**

1. What are the optimal dosages and routes of administration of molecular hydrogen for achieving radioprotection?
2. Does the radioprotective efficacy of molecular hydrogen vary depending on the type of radiation (e.g. gamma rays, X-rays or particle radiation)?
3. Does the protective effect of molecular hydrogen remain effective under conditions of long-term exposure to low-dose radiation, and are there any potential side effects?

## Data Availability

*Not applicable.*

## References

[1] Kokhan VS, Dobynde MI (2023). The effects of galactic cosmic rays on the central nervous system: from negative to unexpectedly positive effects that astronauts may encounter. Biology (Basel).

[2] Ohnishi T (2016). Life science experiments performed in space in the ISS/Kibo facility and future research plans. J Radiat Res.

[3] Giovanetti A, Tortolici F, Rufini S (2020). Why do the cosmic rays induce aging?. Front Physiol.

[4] Rochette L, Zeller M, Cottin Y, Vergely C (2021). Antitumor activity of protons and molecular hydrogen: underlying mechanisms. Cancers (Basel).

[5] Ichikawa Y, Satoh B, Hirano SI, Kurokawa R, Takefuji Y, Satoh F (2020). Proposal of next-generation medical care “Mega-hydrogen Therapy”. Med Gas Res.

[6] Luoni F, Boscolo D, Fiore G (2022). Dose attenuation in innovative shielding materials for radiation protection in space: measurements and simulations. Radiat Res.

[7] Fontana F, Limonta P (2021). The multifaceted roles of mitochondria at the crossroads of cell life and death in cancer. Free Radic Biol Med.

[8] Zhang S, Xin W, Anderson GJ (2022). Double-edge sword roles of iron in driving energy production versus instigating ferroptosis. Cell Death Dis.

[9] Jay-Gerin JP (2025). Fundamentals of Water Radiolysis. Encyclopedia.

[10] Katsura M, Cyou-Nakamine H, Zen Q (2016). Effects of chronic low-dose radiation on human neural progenitor cells. Sci Rep.

[11] United Nations Scientific Committee on the Effects of Atomic Radiation (2000). Sources and effects of ionizing radiation, United Nations Scientific Committee on the Effects of Atomic Radiation (UNSCEAR) 2000 report, Volume I Report to the general assembly, with scientific annexes - sources. United Nations Scientific Committee on the Effects of Atomic Radiation (UNSCEAR) Reports.

[12] Valentin J (2007). Annals of the ICRP publication 103 the 2007 Recommendations of the International Commission on Radiological Protection. ICRP.

[13] Lochana WVAS, Welagedara MLD (2025). Health physics implications of space radiation for astronauts. KJMS.

[14] Pacini AA (2017). Cosmic rays: bringing messages from the sky to the Earth’s surface. Rev Bras Ensino Fís.

[15] Wilson CTR (1997). On a method of making visible the paths of ionising particles through a gas. Proc R Soc Lond A.

[16] Zheng Z, Su J, Bao X (2023). Mechanisms and applications of radiation-induced oxidative stress in regulating cancer immunotherapy. Front Immunol.

[17] Yoshida Y, Tanaka S (2022). Deciphering the biological enigma-genomic evolution underlying anhydrobiosis in the phylum tardigrada and the chironomid Polypedilum vanderplanki. Insects.

[18] Li L, Ge Z, Liu S (2024). Multi-omics landscape and molecular basis of radiation tolerance in a tardigrade. Science.

[19] Khanna KK, Jackson SP (2001). DNA double-strand breaks: signaling, repair and the cancer connection. Nat Genet.

[20] Guo J, Zeitlin C, Wimmer-Schweingruber RF (2021). Radiation environment for future human exploration on the surface of Mars: the current understanding based on MSL/RAD dose measurements. Astron Astrophys Rev.

[21] Vieira CLZ, Janot-Pacheco E, Lage C (2018). Long-term association between the intensity of cosmic rays and mortality rates in the city of Sao Paulo. Environ Res Lett.

[22] Luckey TD (2006). Radiation hormesis: the good, the bad, and the ugly. Dose Response.

[23] Yamamoto H, Ichikawa Y, Hirano SI, Sato B, Takefuji Y, Satoh F (2021). Molecular hydrogen as a novel protective agent against pre-symptomatic diseases. Int J Mol Sci.

[24] Camell CD, Yousefzadeh MJ, Zhu Y (2021). Senolytics reduce coronavirus-related mortality in old mice. Science.

[25] Liang J, Ziegler JD, Jahraus B (2020). Piperlongumine acts as an immunosuppressant by exerting prooxidative effects in human T cells resulting in diminished T(H)17 but enhanced T(reg) differentiation. Front Immunol.

[26] Zhang X, Zhang S, Liu X (2018). Oxidation resistance 1 is a novel senolytic target. Aging Cell.

[27] Yanagihara T, Arai K, Miyamae K (2005). Electrolyzed hydrogen-saturated water for drinking use elicits an antioxidative effect: a feeding test with rats. Biosci Biotechnol Biochem.

[28] Hirano SI, Ichikawa Y, Sato B, Yamamoto H, Takefuji Y, Satoh F (2021). Molecular hydrogen as a potential clinically applicable radioprotective agent. Int J Mol Sci.

[29] Hirano SI, Aoki Y, Li XK, Ichimaru N, Takahara S, Takefuji Y (2021). Protective effects of hydrogen gas inhalation on radiation-induced bone marrow damage in cancer patients: a retrospective observational study. Med Gas Res.

[30] Rosenfeld L, Hoyer U (1981). II. The trilogy: on the constitution of atoms and molecules. Niels bohr collected works.

[31] Cole AR, Sperotto F, DiNardo JA (2021). Safety of prolonged inhalation of hydrogen gas in air in healthy adults. Crit Care Explor.

[32] Kang KM, Kang YN, Choi IB (2011). Effects of drinking hydrogen-rich water on the quality of life of patients treated with radiotherapy for liver tumors. Med Gas Res.

[33] Kuo DY, Wang YC, Chou PH, Lai CW, Tung FI, Liu TY (2024). Therapeutic potential of hydrogen as a radioprotective agent for the prevention of radiation dermatitis. Antioxidants (Basel).

[34] Anderson AW (1956). Studies on a radioresistant micrococcus. 1. Isolation, morphology, cultural characteristics, and resistance to γ radiation. Food Technol.

[35] White O, Eisen JA, Heidelberg JF (1999). Genome sequence of the radioresistant bacterium Deinococcus radiodurans R1. Science.

[36] Maqbool I, Ponniresan VK, Govindasamy K, Prasad NR (2020). Understanding the survival mechanisms of Deinococcus radiodurans against oxidative stress by targeting thioredoxin reductase redox system. Arch Microbiol.

[37] Liu F, Li N, Zhang Y (2023). The radioresistant and survival mechanisms of Deinococcus radiodurans. Radiat Med Prot.

[38] Daly MJ (2009). A new perspective on radiation resistance based on Deinococcus radiodurans. Nat Rev Microbiol.

[39] Xue D, Liu W, Chen Y (2019). RNA-seq-based comparative transcriptome analysis highlights new features of the heat-stress response in the extremophilic bacterium Deinococcus radiodurans. Int J Mol Sci.

[40] Jolivet E, Corre E, L’Haridon S, Forterre P, Prieur D (2004). Thermococcus marinus sp. nov. and Thermococcus radiotolerans sp. nov., two hyperthermophilic archaea from deep-sea hydrothermal vents that resist ionizing radiation. Extremophiles.

[41] Albuquerque L, Simões C, Nobre MF (2005). Truepera radiovictrix gen. nov., sp. nov., a new radiation resistant species and the proposal of Trueperaceae fam. nov. FEMS Microbiol Lett.

[42] Ferreira AC, Nobre MF, Moore E, Rainey FA, Battista JR, da Costa MS (1999). Characterization and radiation resistance of new isolates of Rubrobacter radiotolerans and Rubrobacter xylanophilus. Extremophiles.

[43] Collins MD, Hutson RA, Grant IR, Patterson MF (2000). Phylogenetic characterization of a novel radiation-resistant bacterium from irradiated pork: description of Hymenobacter actinosclerus sp. nov. Int J Syst Evol Microbiol.

[44] Russell G (2025). Theoretical evaluation of the biological activity of hydrogen. Med Gas Res.

[45] Zhou Z, Zhang W, Chen M (2011). Genome-wide transcriptome and proteome analysis of Escherichia coli expressing IrrE, a global regulator of Deinococcus radiodurans. Mol Biosyst.

[46] Leone L, Sgueglia G, La Gatta S, Chino M, Nastri F, Lombardi A (2023). Enzymatic and bioinspired systems for hydrogen production. Int J Mol Sci.

[47] Ignatov I, Popova TP, Vassileva P, Marinov YG, Iliev MT (2025). Hot mineral water as a medium for molecular hydrogen reactions in the primordial hydrosphere for the origin of life. Hydrogen.

[48] Ichikawa Y, Yamamoto H, Hirano SI, Sato B, Takefuji Y, Satoh F (2023). The overlooked benefits of hydrogen-producing bacteria. Med Gas Res.

[49] Theodorakopoulos N, Bachar D, Christen R, Alain K, Chapon V (2013). Exploration of deinococcus-thermus molecular diversity by novel group-specific PCR primers. Microbiologyopen.

[50] Khaksarighiri S, Guo J, Wimmer-Schweingruber R, Narici L (2021). An easy-to-use function to assess deep space radiation in human brains. Sci Rep.

[51] Tekutskaya EE, Ryabova IS (2021). Reactive oxygen species, DNA damage and solar-Earth communications. IOP Conf Ser Earth Environ Sci.

[52] Ruff RL (2008). Mild traumatic brain injury in U.S. soldiers returning from Iraq. N Engl J Med.

[53] Paladini MS, Feng X, Krukowski K, Rosi S (2021). Microglia depletion and cognitive functions after brain injury: From trauma to galactic cosmic ray. Neurosci Lett.

[54] Rienecker KDA, Paladini MS, Grue K, Krukowski K, Rosi S (2021). Microglia: ally and enemy in deep space. Neurosci Biobehav Rev.

[55] Rahman MH, Bajgai J, Fadriquela A (2021). Redox effects of molecular hydrogen and its therapeutic efficacy in the treatment of neurodegenerative diseases. Processes.

[56] Hirano SI, Ichikawa Y, Sato B, Satoh F, Takefuji Y (2020). Hydrogen is promising for medical applications. Clean Technol.

